# Research on Broadband Matching Method for Capacitive Micromachined Ultrasonic Transducers Based on PDMS/TiO_2_ Particles

**DOI:** 10.3390/mi13111827

**Published:** 2022-10-26

**Authors:** Bizhen Gao, Sai Zhang, Changde He, Renxin Wang, Yuhua Yang, Licheng Jia, Zhihao Wang, Yang Wu, Shumin Hu, Wendong Zhang

**Affiliations:** 1 State Key Laboratory of Dynamic Measurement Technology, School of Instrumentand Electronics, North University of China, Taiyuan 030051, China; 2 Department of Physics, The Institute of Ultrasonic Testing, Jiangsu University, Zhenjiang 212013, China

**Keywords:** capacitive micromachined ultrasonic transducer (CMUT), acoustic communication, acoustic matching layer, 0–3 composite materials, broadband

## Abstract

The study of impedance matching between a transducer and its working medium is an important part of acoustic transducer design. The traditional quarter wavelength matching (Q-matching) scheme is not suitable for broadband capacitive micromachined ultrasonic transducers. To mitigate this issue, a 0–3 composite broadband matching layer based on polydimethylsiloxane (PDMS) substrate/TiO_2_ particles is designed to achieve electrical insulation and efficient acoustic energy transfer of underwater capacitive micromachined ultrasonic transducer (CMUT) devices. In this work, the coherent potential approximation model is used to analyze the properties of 0–3 composite materials. Samples are prepared for performance testing to determine the proportion of TiO_2_ particles that enable the 0–3 composite materials to have the same longitudinal acoustic impedance as water. The CMUT device is packaged by a spin coating and pouring process, and its performance tests are carried out. The experimental results show that the central frequency of the transducer remains at 1.74 MHz, the −6 dB fractional bandwidth increases from 97.3% to 100.3%, the 3 dB directional main beam width increases from 8.3° to 10.3°, the side lobes decrease significantly, and the device has good reception sensitivity. These values imply that the 0–3 composite material has good matching performance, and this matching scheme has the advantages of high efficiency and wide bandwidth. This broadband matching method endows CMUTs with great advantages in underwater detection systems, and it facilitates underwater ultrasonic imaging of CMUT.

## 1. Introduction

Ultrasonic imaging is based on the reflection, refraction and transmission of ultrasound in different media [1,2,3]. An ultrasonic transducer is an acoustic device that is used to transmit and receive ultrasonic waves. It is the core device in ultrasonic applications and realizes the mutual conversion between electrical energy and mechanical vibration energy [4,5,6]. It is widely used in medical ultrasonic imaging, industrial ultrasonic nondestructive testing and underwater acoustic engineering [7,8,9]. With the development of micro-electro-mechanical systems (MEMS), the capacitive micromachined ultrasonic transducer (CMUT) based on MEMS micro machining technology has been attracting an increasing amount of attention. It has significant advantages that traditional piezoelectric transducers do not have, such as a simple and flexible structure, high sensitivity, high electro-mechanical conversion efficiency, high resolution, wide bandwidth, and good consistency. It also has the potential to easily form large arrays and integrate with electronic devices [10,11,12,13,14,15,16,17,18]. In addition, it can match better with the acoustic transmission medium because its diaphragm has a lower acoustic impedance [19,20], providing favorable conditions for ultrasonic imaging.

Water is often used in ultrasonic imaging as a coupling agent to achieve the efficient transmission of ultrasonic energy. However, CMUT devices cannot be directly used in the water environment because the surface of the diaphragm is covered with electrodes. In order to protect the CMUT devices from the external environment and mechanical damage, it is necessary to package them. As the medium of ultrasonic propagation between the CMUT device and the water environment, the packaging structure needs to solve the impedance matching problem. A large number of studies have shown that the impedance matching imbalance adversely affects the acoustic radiation efficiency, working bandwidth and directivity of the transducer [21,22]. Therefore, the selection of matching layer and packaging design is critical to the performance of ultrasonic transducers.

**Figure 1:**
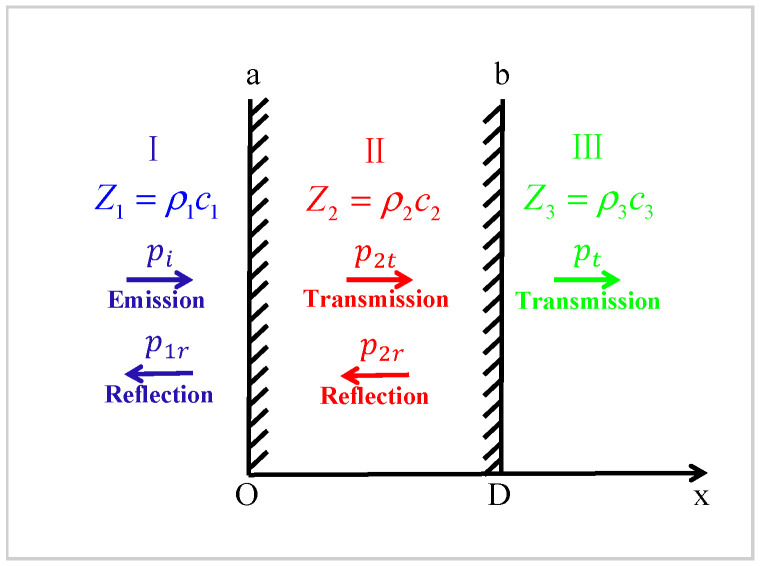
Wave propagation through interfaces between different media.

The traditional quarter wavelength matching (Q-matching) [22] theory regards two media as infinite ideal fluids with different characteristic impedances. A matching layer is inserted between two media in order to achieve full transmission of acoustic energy. Figure 1 schematically shows the propagation of sound waves through the interface of two media, including the matching layer. The acoustic impedance of the intermediate matching layer should meet the following conditions:

(1) $$Z_{2}=\sqrt{Z_{1}Z_{3}}$$

(2) $$D=\left(2n-1\right)\frac{1}{4}\lambda_{2}\left(n=1,2,3,\ldots ,N\right)$$

This means that lossless sound transmission can be achieved when the acoustic impedance of the matching layer is the geometric average of the acoustic impedance of the two propagation media, and its thickness (D) is an odd multiple of the quarter wavelength of the matching layer medium. Although this classical matching scheme improves the impedance mismatch between the transducer and the working medium to some extent, the following problems still exist: 

(1) The bandwidth is narrow. Full acoustic transmission can be achieved only when the acoustic impedance and thickness satisfy (2).

(2) It can only have a high relative pulse echo sensitivity at the operating frequency. The relative pulse echo sensitivity (or bidirectional insertion loss) of the transducer represents its energy conversion efficiency. It is the ratio between the output power and the input power of the transducer, which can be simplified as the ratio between the echo signal voltage and the excitation signal voltage.

It can be gathered that the traditional matching theory has obvious performance limitations on CMUT, which is a new transducer with broadband. Therefore, the development of broadband materials has important application value for the impedance matching of acoustic ultrasonic imaging.

At present, most CMUT packaging materials that work in water are polyurethane, polydimethylsiloxane (PDMS) and parylene-C. Zhang et al. [23] studied the underwater array packaging method based on the requirements of capacitive micromachined ultrasonic transducers underwater applications. The authors further selected sound transmission liquid (silicone oil) and sound transmission film (polyurethane) for packaging, which are corrosion resistant and easy to process. However, neither research nor optimization of the packaging method was conducted, and only a preliminarily underwater detection application was realized. He et al. [24] covered the CMUT-membrane with a 5 μm thick parylene-C layer to meet the waterproofing requirements of underwater measurements. Hsu et al. [25] used parylene-C to form a 2 μm thick protective layer on the CMUT-membrane and pointed out the key problems. The time for the parylene-C coating to ensure electrical insulation in an aqueous solution was 14 days, and it exhibited poor long-term stability. Nikoozadehe et al. [26] coated the CMUT array with a layer of polydimethylsiloxane (PDMS) having a thickness of 180 μm to electrically isolate the device for imaging in water and tissues. Mette et al. [27] used PDMS with a thickness of about 900 μm to package the CMUT. The report showed that the center frequency of the packaged CMUT was shifted from 4.5 to 4.1 MHz during transmission. During the reception, the center frequency was reduced from 4.4 to 3.9 MHz, and the transmission pressure and reception sensitivity were reduced by about 30%.

Existing research has shown that the matching layer of specific acoustic impedance can also be obtained by fabricating composite materials, and the continuous and wide range acoustic impedance design can be achieved by adding or changing the filler composition and its proportion [28,29,30]. Shaoping et al. [31] selected epoxy resin and aluminum powder to prepare a composite matching layer, and they expanded the bandwidth of the transducer covering this matching layer. Park et al. [29] prepared the acoustic matching layer film using epoxy resin with different volume fractions of aluminum oxide and tungsten powder. Using the optimized acoustic matching layer, the maximum intensity and fractional bandwidth of the ultrasonic transducer were increased by 10% and 37%, respectively, which successfully verified the effectiveness of the optimized matching layer. Guillermic et al. [32] prepared the 0–3 composite material by mixing polydimethylsiloxane (PDMS) and titanium dioxide particles. The optimized composite material had the same longitudinal acoustic impedance as water, and its acoustic reflection coefficient was basically zero in a wide frequency range (0.5 to 6 MHz).

Based on this behavior, we propose to prepare 0–3 composite materials through PDMS and TiO_2_ particles for underwater CMUT broadband matching layers, and we carry out underwater CMUT packaging and performance tests. We study the influence of this 0–3 composite material on the CMUT performance so as to provide technical support for the independent research and development of underwater exploration ultrasound equipment.

## 2. Working Principle of CMUT

**Figure 2:**
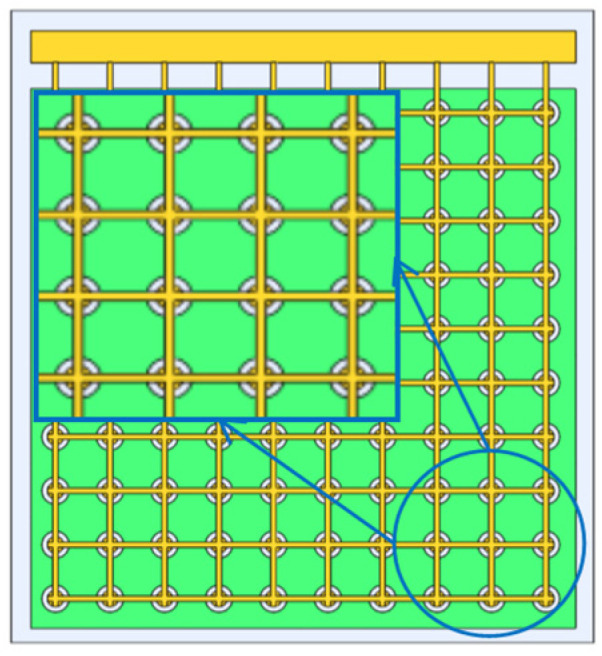
Schematic view of capacitive micromachined ultrasonic transducer array element.

**Figure 3:**
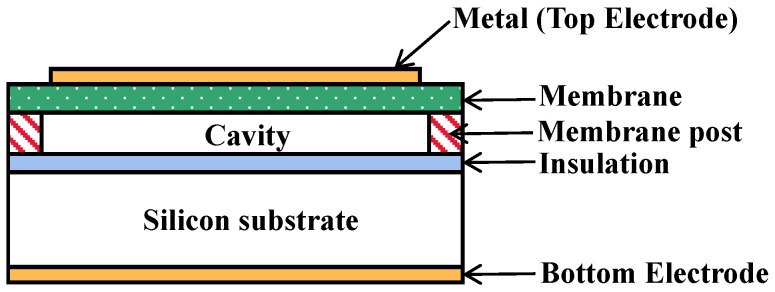
Schematic cross-section of CMUT cells.

As Figure 2 shows, a single CMUT array element is composed of multiple micro cells arranged in parallel in a regular fashion. Figure 3 shows the schematic diagram of the cross-section of the micro cell. From top to bottom, this structure consists of (1) the aluminum metal electrode, (2) the vibration film made of the top silicon of the SOI sheet, (3) the silicon oxide isolation layer, (4) the closed vacuum cavity etched by the silicon oxide, (5) the silicon oxide insulation layer, (6) the silicon substrate and (7) the aluminum metal electrode. The working mode of CMUT cannot be separated from the DC bias voltage. In the working mode, the DC bias voltage is applied to the upper and lower electrodes, and the generated electrostatic force will pull the film vertically in the direction of the downward electrode. However, due to the reverse recovery force of the film itself, the film will soon stop moving and reach the equilibrium state. If the required frequency of AC excitation voltage is applied again at this moment, the film will reciprocate vibration and radiate ultrasonic waves of the corresponding frequency. In the equilibrium state, the external sound pressure changes on the film will make the film vibrate and then change the capacitance of the cavity. Consequently, it will generate a weak current signal. After trans-resistance amplification and other processing, the circuit can realize voltage signal reception [23,33,34].

## 3. Performance Analysis of the 0–3 Composite Material

### 3.1. Prediction of Properties of the 0–3 Composite Material

The acoustic effective properties of composites can be predicted by the model. The analysis for the case of wavelengths larger than a single particle is performed using an effective medium theory, such as the coherent potential approximation model. For elastic composites, the effective composite shear modulus ($G_{eff}=G_{eff}^{\prime }+iG_{eff}^{\prime \prime }$, where $G_{eff}^{\prime }$ and $G_{eff}^{\prime \prime }$ are the real and imaginary parts of the composite shear modulus) and the effective composite longitudinal modulus ($M_{eff}=M_{eff}^{\prime }+iM_{eff}^{\prime \prime }$, where $M_{eff}^{\prime }$ and $M_{eff}^{\prime \prime }$ are the real and imaginary parts of the composite longitudinal modulus) of the medium can be calculated according to the elastic modulus of the two components [35,36,37], respectively.

(3) $$M_{eff}=\frac{M_{0}\left[4\left(G_{0}-G_{1}\right)+3M_{1}\right]}{3pM_{0}+\left(1-p\right)\left[4\left(G_{0}-G_{1}\right)+3M_{1}\right]}-\frac{4}{3}\left(G_{0}-G_{eff}\right)$$

(4) $$A{\left(\frac{M_{eff}}{M_{0}}\right)}^{2}+B\left(\frac{M_{eff}}{M_{0}}\right)+C=0$$

where subscripts 1 and 0 represent the particle and matrix medium, respectively, G_0_ and M_0_ represent the composite shear and longitudinal moduli of the matrix, respectively, G_1_ and M_1_ represents the composite shear and longitudinal moduli of the particle, respectively, and *p* represents the volume fraction of the filler. The parameters *A*, *B*, and *C* are functions of *M*_0_, *M*_1_, *G*_0_ and *G*_1_.

The effective density can be found by the mixture law as follows:

(5) $$\rho_{eff}=\rho_{0}\left(1-p\right)+\rho_{1}p$$

where $\rho_{0}$ denotes the density of substrate and $\rho_{1}$ denotes the density of particles.

The effective longitudinal phase velocity (*v*_eff_) [32] can be obtained from the relationship between longitudinal modulus, velocity and decay as

(6) $$M_{eff}^{\prime }=\rho_{eff}v_{eff}{}^{2}\frac{1-{\left(\frac{\alpha v_{eff}}{2\omega }\right)}^{2}}{{\left[1+{\left(\frac{\alpha v_{eff}}{2\omega }\right)}^{2}\right]}^{2}}$$

(7) $$M_{eff}^{\prime \prime }=\frac{\rho_{eff}v_{eff}{}^{2}\left(\frac{\alpha v_{eff}}{2\omega }\right)}{{\left[1+{\left(\frac{\alpha v_{eff}}{2\omega }\right)}^{2}\right]}^{2}}$$

where α denotes medium attenuation, and the angular frequency is given by ω=2πf.

Using the low attenuation approximation ($\alpha \ll 2\omega /\nu_{eff}$), we obtain

(8) $$v_{eff}=\sqrt{\frac{M_{eff}^{\prime }}{\rho_{eff}}}$$

From the above expression, the effective impedance (*Z*_eff_) can be obtained as

(9) $$Z_{eff}=\rho_{eff}v_{eff}=\sqrt{M_{eff}^{\prime }\rho_{eff}}$$

**Figure 4:**
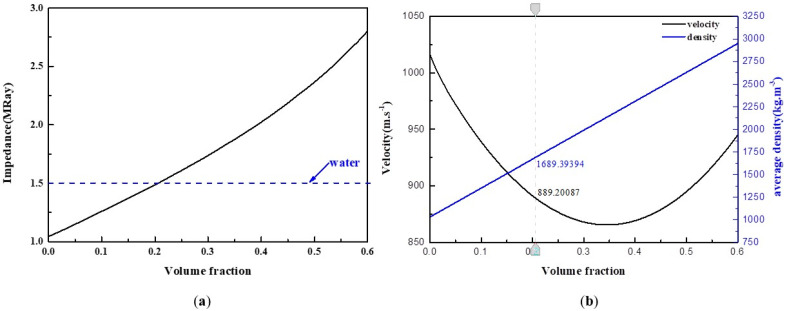
(**a**) The functional relationship between acoustic impedance and volume fraction of composite materials. (**b**) Velocity and density of composite PDMS-TiO_2_ materials as a function of volume fraction of TiO_2_ particles.

Table 1 shows the properties of the PDMS matrix and TiO_2_ particles (as described in references [32,38]). The parameters are used in the coherent potential approximation model to predict the phase velocity and density of the 0–3 composite material, and we explore the functional relationship with the volume fraction of TiO_2_ particles. This provides a theoretical basis for the preparation of a suitable broadband matching layer. Figure 4a,b show the predicted phase velocity and density obtained using the parameters in Table 1 as a function of TiO_2_ particle volume fraction. It was determined based on the model prediction and the known water impedance (1.5 MRay) that the TiO_2_ particles with a volume fraction of 20.6% dispersed in the PDMS matrix would provide a good impedance match with a composite velocity of 889 m/s and a density of 1690 kg/m^3^.

### 3.2. Preparation of 0–3 Composite Material Sample

**Figure 5:**
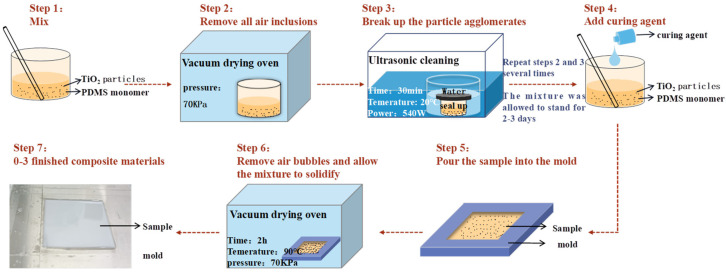
Preparation setup of the 0–3 composite material sample.

The 0–3 composite sample consisted of PDMS prepolymers, which contained titanium dioxide particles. RTV 615 is a low viscosity, high strength, addition and curing medical PDMS prepolymer, including monotherapy agent (A) and curing agent (B). We used RTV 615 polydimethylsiloxane and TiO_2_ particles with a radius of 20 nm to prepare the 0–3 composite material, as shown in Figure 5. The preparation process is as follows:

The monomeric part of PDMS was poured into a beaker. An appropriate amount of titanium dioxide powder was added to it. The mixture was then stirred using a stirring bar to mix thoroughly and placed in a vacuum drying oven to remove all air inclusions (steps 1 and 2).

The mixture was placed in the ultrasonic cleaning machine for 30 min of ultrasonic vibration (step 3) to break the particle aggregates and fully mix the titanium dioxide powder. The above steps were repeated several times if necessary, leaving the sample for two to three days to optimize the uniformity of the mixture.

The stainless steel mold was prepared and the curing agent was added to the mixture. The mixture was poured into the flat stainless steel mold. The mold was then placed into a vacuum drying oven for further evacuation (steps 4 and 5).

The samples were placed in a drying oven at 90 °C for 120 min to solidify them (step 6), and the finished products are shown in Figure 5 (step 7).

### 3.3. Performance Test of 0–3 Composite Material

**Figure 6:**
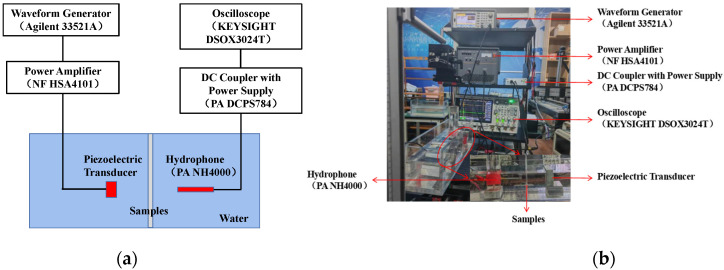
(**a**) Schematic of measurement setup of 0–3 composite material performance. (**b**) Photograph of the measurement setup.

In order to evaluate the performance of the 0–3 composite material, a test-friendly platform was used, and the layout was set up, as shown in Figure 6. A standard transducer (PZT) was used as the ultrasonic transmitter, and the broadband needle hydrophone (NH4000, Precision Acoustics Ltd., Dorchester, UK) was used as the receiver. The two sensors were placed face to face and parallel to each other in the tank to facilitate testing, and the different samples (concentration, thickness) were evenly clamped in the middle of the acrylic plate and inserted vertically into the tank. The Agilent 33521A arbitrary waveform generator was used as the AC signal source on the transmitter side, and the DC coupler with power supply (PA DCPS784) was used on the receiver side. The KEYSIGHT DSOX3024T digital oscilloscope was used to measure and record the transmitted and received waveforms. A waveform generator was used to generate five cycles of AC burst signals, and time-dependent transmission signals and reference signals were collected. The reference signal was measured with the sample removed without changing the settings, where the sensor remained in the same position.

**Figure 7:**
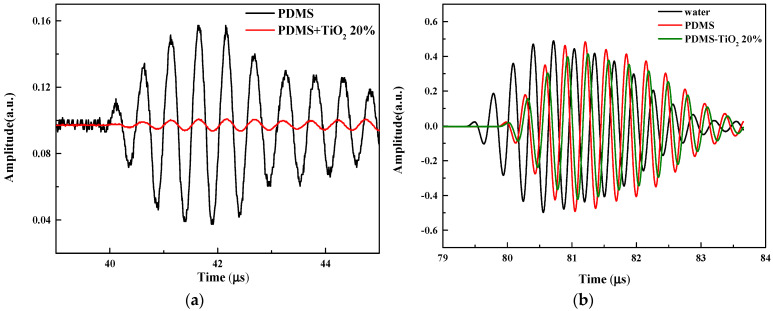
Typical acoustic signals passing through pure PDMS and PDMS with 20% of TiO_2_ particles. (**a**) Reflected signals; (**b**) Transmitted signals.

In order to solve the problem of matching between CMUT and water impedance, the 0–3 composite material (PDMS loaded with 20% TiO_2_) with similar water impedance was selected for the performance test according to the prediction results of the model. Pure PDMS was used as a reference to maintain a consistent sample thickness (1.2 mm). Figure 7a shows the signal received after a 3 MHz acoustic signal was reflected from pure PDMS (black line) and PDMS loaded with 20% titanium dioxide (red line). In the pure PDMS case, the echo signal can be clearly seen from the reflection of the “water–PDMS” interface. However, almost no reflected signal is observed in the case of PDMS loaded with 20% TiO_2_. This reflects that compared with pure PDMS, the acoustic impedance of PDMS loaded with 20% titanium dioxide is closer to that of water.

Figure 7b shows the transmitted signals after 3 MHz acoustic signals pass through water, pure PDMS and PDMS-TiO_2_ 20%, respectively. It can be clearly observed that the time delay between the signals increases, which is related to the decreasing in sound speed. Using the measured “flight time” of sound wave propagating from the transmitter to the receiver and the known propagation distance and matching layer thickness, the sound speed of PDMS-TiO_2_ 20% is calculated as 892.5 m/s. The results are basically consistent with the theory, which confirms the reliability of the model. Compared with the reference signal, the transmission signal amplitudes of the pure PDMS sample and PDMS-TiO_2_ sample are reduced. In addition, the transmission signal amplitude of the PDMS-TiO_2_ sample is lower than that of the pure PDMS, which proves that mixing titanium dioxide particles in PDMS increases the attenuation. This reflects that PDMS with mixed titanium dioxide particles will bring energy loss. We need to study the attenuation law of PDMS with mixed titanium dioxide particles to reduce the sound energy loss as much as possible.

**Figure 8:**
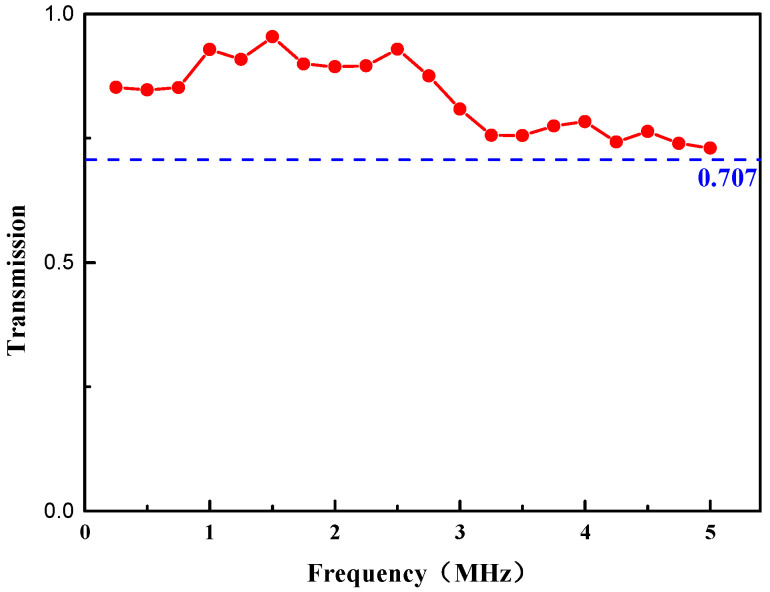
Frequency response of the transmission coefficients of the matching layer.

The underwater acoustic transmission experiment was carried out in the frequency band range of 0.25–5 MHz. The thickness of the matching layer was 1.2 mm, and the proportion of TiO_2_ particles was 20%. Figure 8 shows the frequency dependence of the matching layer observed in the experiment. It is observed that during the broadband transmission, the matching layer has high transmission coefficients in the measurement frequency range of 0.25–5 MHz. In other words, the 0–3 composite material can achieve full transmission of sound waves in the range of 0.25–5 MHz with broadband matching characteristics.

The attenuation of 0–3 composite materials with different thickness values was measured in the 1–4 MHz frequency band. The TiO_2_ particles accounted for 20% of the volume. The measured received voltage data were analyzed and converted into acoustic attenuation values. The corresponding formula is as follows:

(10) $$TL=20log\left(\frac{V_{i}}{V_{t}}\right)$$

where *TL* is the insertion loss of the matching layer, *V_i_* is the amplitude of the received signal when there is no matching layer, and *V_t_* is the amplitude of the received signal when there is a matching layer.

**Figure 9:**
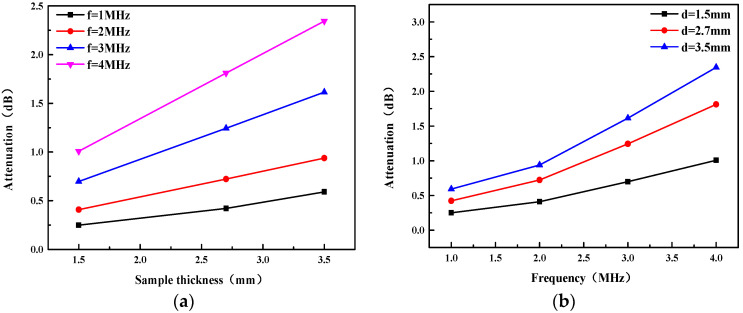
(**a**) Attenuation at representative frequencies as a function of sample thickness; (**b**) Attenuation as a function of frequency for samples with different thickness values.

The attenuation as a function of frequency and sample thickness (D) was studied by quantitative analysis of the measured data. Figure 9a shows that the attenuation increases approximately linearly with sample thickness, and Figure 9b shows that the attenuation increases as the frequency increases. Unlike velocity, the attenuation varies considerably with frequency, and it is more pronounced at higher frequencies. Nevertheless, the attenuation is low enough over the whole range of frequency bands studied. It is necessary to control the thickness of the matching layer during the CMUT packaging process in order to reduce energy loss. This ensures that the matching layer is thin enough but not easily damaged.

## 4. CMUT Encapsulation and Testing

### 4.1. CMUT Encapsulation Steps

**Figure 10:**
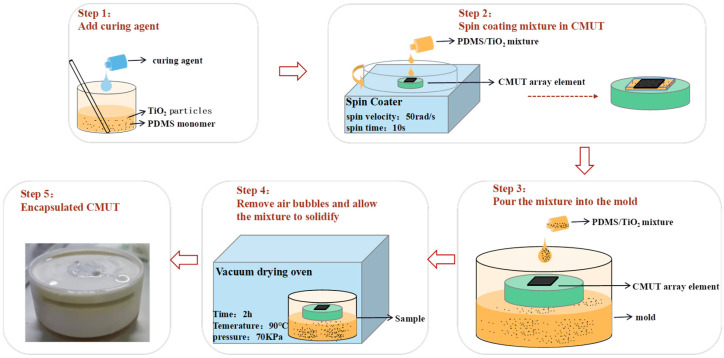
Preparation setup of CMUT packaging.

The CMUT device packaging experiment was carried out. Table 2 shows the CMUT device parameters used in the experiment. The CMUT should be completely sealed in order to protect the CMUT from external environmental and mechanical damage. In addition, due to the attenuation caused by the 0–3 composite material, it was necessary to control the preparation thickness. The packaging steps for covering the surface of CMUT chip with 0–3 composite material of 200 μm thickness are shown in Figure 10. In the first step, the 0–3 composite material was prepared, and the surface of the CMUT membrane was covered with the rotary coating process [39]. The CMUT device was placed in the homogenizer, and an appropriate amount of 0–3 composite material was dropped in the center of the membrane. The speed and time of the homogenizer were adjusted to 50 rad/s and 10 s, respectively, and it was rotated to evenly spread the 200 μm thick material on the surface of the CMUT membrane. The equipment was placed into the vacuum drying oven for evacuation and curing. In the second step, the mold was customized, and the CMUT (except for the surface of the CMUT-membrane) was completely encapsulated by filling the mold with the composite material. Figure 10 shows the CMUT device with the 0–3 composite material coating (step 5).

### 4.2. CMUT Performance Test

Acoustic tests were performed on the encapsulated CMUT element to evaluate its performance. The axial acoustic field characteristics of the CMUT element were tested. Measurements were obtained using the CMUT element as the ultrasonic transmitter and the hydrophone (NH4000, Precision Acoustics Ltd., Dorchester, UK) as the receiver. At the transmitter side, an Agilent 33521A arbitrary waveform generator was used as the AC signal source. Five continuous sinusoidal pulse excitation signals with an amplitude of 10 Vpp and a frequency of 3 MHz were considered as the excitation parameters. A 20V DC bias voltage was applied from a power amplifier (HSA4101, NF Corporation, Kanagawa, Japan) to drive the CMUT. A DC coupler with power supply (PA DCPS784) was connected to the oscilloscope at the receiver side for measuring and recording the transmit and receive waveforms.

**Figure 11:**
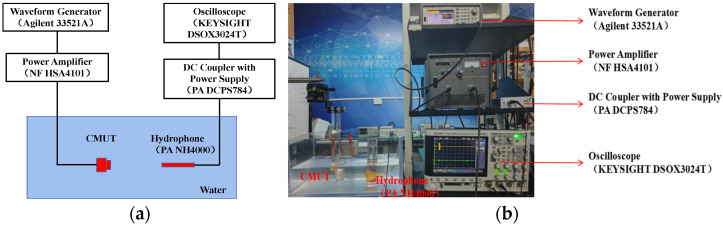
(**a**) Schematic of measurement setup of the CMUT transmitting performance. (**b**) Photograph of the measurement setup.

**Figure 12:**
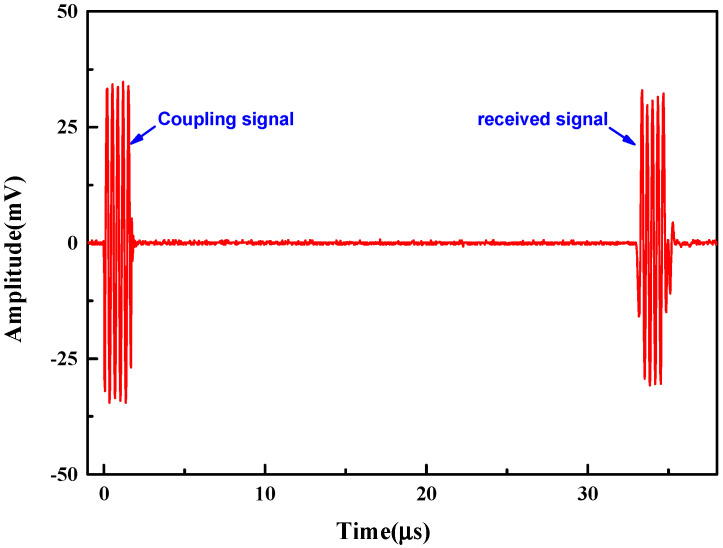
Response waveforms of the hydrophone when the CMUT was driven by the excitation waveform and DC bias voltages.

To facilitate the test, the CMUT was mounted on the test fixture. Subsequently, it was mounted on the XYZ positioner to control the movement of the fixture, and the bracket was used to fix the needle hydrophone, as shown in Figure 11 for the test device. Figure 12 shows the output response of the needle hydrophone when the CMUT was driven by a sinusoidal pulse voltage signal. The received signal waveform is intact. The result shows that the encapsulated CMUT can work normally in the water, and the encapsulation does not adversely affect the waveform.

**Figure 13:**
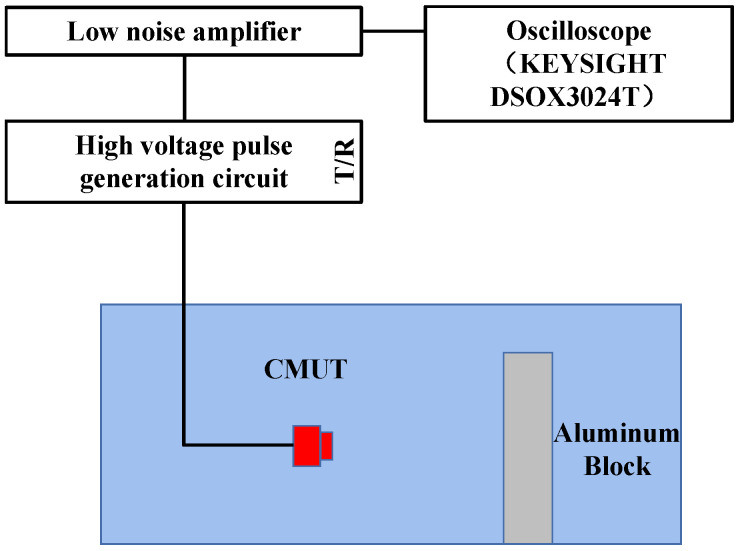
Schematic diagram of pulse echo device test.

The pulsed echo method was used to test the bandwidth of the CMUT device. The CMUT device transmitted the ultrasonic signals and received the echo signals reflected by aluminum blocks. The distance between the CMUT and the aluminum block was 5 cm. Figure 13 shows the test device. The Olympus 5073PR ultrasonic transducer bandwidth test commercial instrument was used as the ultrasonic pulse transmitter receiver. The instrument generated a single narrow pulse voltage signal to drive the CMUT equipment to transmit ultrasonic waves. After receiving the ultrasonic echo signal reflected by the aluminum block, the signal was converted by the low-noise amplifier in the 5073PR instrument and output to the oscilloscope. The corresponding frequency map of the signal was obtained after the Fourier transform of the echo signal.

**Figure 14:**
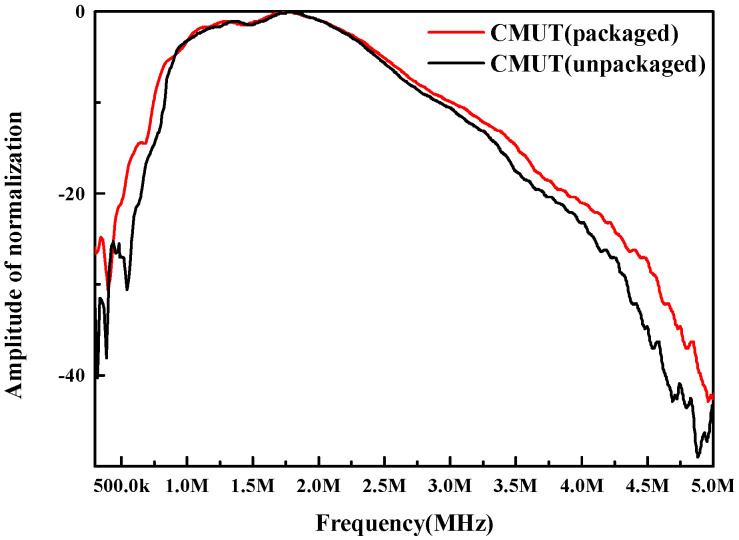
Frequency response function of the CMUT with and without coating.

In the same settings, the unencapsulated CMUT bare chip was tested in silicone oil media as a reference because the impedance of silicone oil is similar to that of water, and the authors had previously used a silicone oil/polyurethane combination to encapsulate the CMUT. As Figure 14 shows, the unpackaged CMUT center frequency was 1.74 MHz and the −6 dB fractional bandwidth was 97.3%, while the packaged CMUT center frequency was 1.74 MHz and the −6 dB fractional bandwidth was 100.3%. The center frequency did not shift, and the bandwidth increased by 4.3%. These results show that the package scheme can play the role of insulation and chip protection, and it can realize the broadband sound transmission of CMUT.

**Figure 15:**
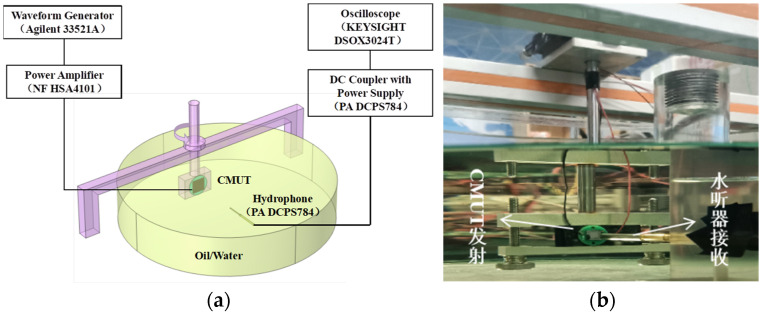
(**a**) Schematic of measurement setup of the CMUT transmitting directivity; (**b**) Photograph of the measurement setup, (left) transmission of CMUT, (right) receiving of the hydrophone.

**Figure 16:**
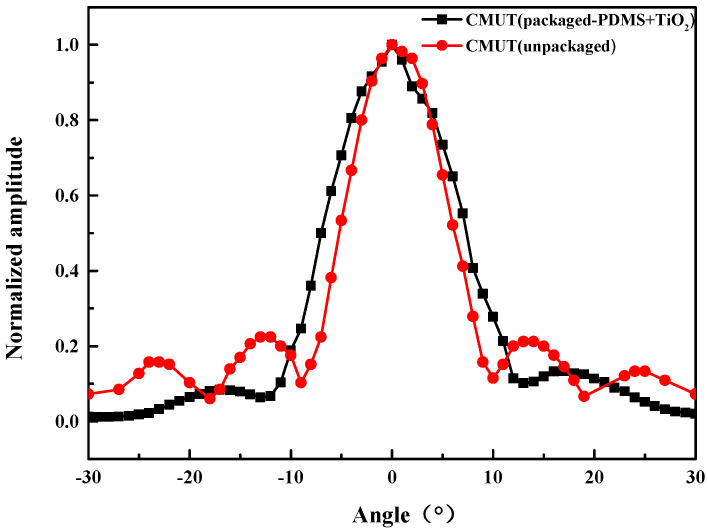
Transmitting directivity of the CMUT with and without coating.

The directivity of the CMUT element was tested, and the test device is shown in Figure 15. The CMUT was used as the transmitter and fixed in water under the precision dividing wheel. The needle hydrophone was used as the receiver and fixed at a distance of 10 cm from the opposite side of the transmitting end. Furthermore, it was kept at the center level with the CMUT. The CMUT rotated with the dial from −30° to 30° with a single step value of 1°. Under the same settings, the test results of the unpackaged CMUT bare chip in silicone oil medium were considered as the reference signals. Figure 16 shows the CMUT directivity test results. It can be observed that the directivity of CMUT has a good symmetry, and the width of the −3 dB main lobe is 8.3°, which is accompanied by side lobe interference. The directivity of the packaged CMUT is symmetrical, and the width of the −3 dB main lobe is 10.3°. The width of the main lobe slightly increased, respectively, compared with the corresponding width for the naked directivity. Compared with the naked directional sidelobe, the sidelobe of the packaged CMUT was obviously suppressed.

**Figure 17:**
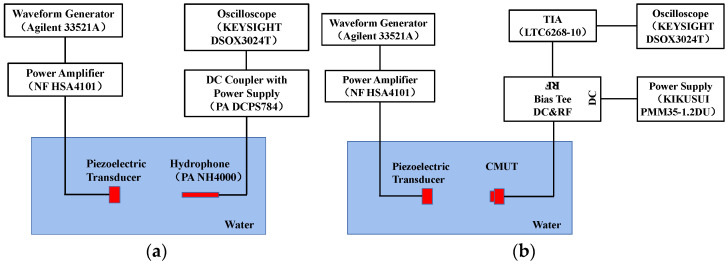
(**a**) Schematic of measurement setup of the CMUT receiving performance; (**b**) Photograph of the measurement setup.

The receiving sensitivity of the CMUT device was tested by the reciprocal calibration method using water as the medium, as shown in Figure 17. A piezoelectric transducer was selected as the emission source for transmitting the ultrasonic waves. A needle hydrophone with known receiving sensitivity received the ultrasonic waves, and an oscilloscope recorded the amplitude of the received signal. Subsequently, the CMUT device to be measured was placed in the same position as the needle hydrophone to receive the ultrasonic waves, and the oscilloscope recorded the output voltage signal after low noise amplification. The piezoelectric transducer was always driven by five sinusoidal pulses with a peak-to-peak amplitude of 50 V. The reception sensitivities of the unpackaged and packaged CMUT were tested and compared at 1 MHz, 2 MHz and 3 MHz, respectively.

Table 3 lists the peak-to-peak amplitude (V_N_) and the receiving sensitivity (S_N_) of the output voltage signal of the needle hydrophone, the peak-to-peak amplitude (V_C_) of the output voltage signal of the unpackaged CMUT, the calculated received sensitivity (S_C_) of the unencapsulated CMUT, and the received sensitivity expressed in decibels (S_CdB_) at different frequencies. Table 4 lists the peak-to-peak amplitude (V_C_) of the output voltage signal of the encapsulated CMUT and the received sensitivity (S_C_) of the encapsulated CMUT, and the received sensitivity (S_CdB_) is expressed in decibels at different frequencies. The receiving sensitivity is calculated using the following expression:

(11) $$S_{C}=\frac{V_{C}}{V_{N}}\times S_{N}$$

The basic value of 0 dB is 1 V/µPa, and the receiving sensitivity is expressed in decibels as

(12) $$S_{CdB}=20logS_{C}-300$$

At 1 MHz, 2 MHz and 3 MHz frequencies, the receiving sensitivities of the unpackaged CMUT were −219.4 dB@1 MHz, −218.5 dB@2 MHz and −220.4 dB@3 MHz, respectively. The received sensitivities of the encapsulated CMUT were −221.2 dB@1 MHz, −220.6 dB@2 MHz, and −224 dB@3 MHz, respectively, where the reference sensitivity was 1 V/μPa. The results show that the encapsulated CMUT still has a good reception sensitivity.

**Figure 18:**
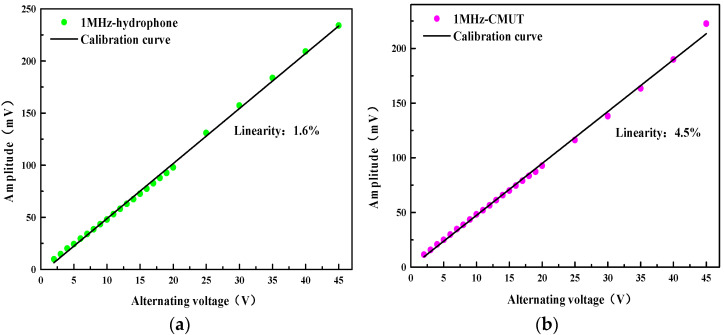
(**a**) Receiving linearity of the hydrophone; (**b**) Receiving linearity of the CMUT with coating.

Next, the CMUT reception performance was evaluated under various AC excitations. The piezoelectric transducer emitted sound waves in water, gradually increasing the amplitude of the transmitted signal, and the transmitted AC signal was changed from 0 to 45 V with a step value of 1 V. The needle hydrophone and CMUT were, respectively, used to receive the signal, and the amplitude curve of the received signal was fitted to analyze and compare the receiving linearity of the hydrophone and CMUT. Figure 18 shows the measurement results. In the range of 0–45 V, the received signal amplitude increases with the increase in the transmitted signal amplitude. The relationship between the received and transmitted signal amplitudes of CMUT and hydrophone is approximately linear. The linearity of CMUT and hydrophone at 1 MHz is calculated. The fitted curve is used as the calibration curve, and the linearity is fitted according to Formula (13). The results are shown in Figure 10, as follows:

(13) $$\delta =\frac{\Delta Y_{max}}{Y}$$

where *Y_max_* is the maximum deviation between the sensor calibration curve and the fitted line, and *Y* is the full-scale output value. The results show that the linearity of the standard hydrophone is 1.6%, and the linearity of the CMUT is 4.5%. The test results show that the packaged CMUT still has good reception linearity.

## 5. Conclusions

In this paper, the CMUT device with an immersion center frequency of 1.74 MHz and fabricated using the silicon wafer bonding process was encapsulated and characterized. The 0–3 composite material based on PDMS and TiO_2_ particles was theoretically analyzed and experimentally evaluated. It achieved full transmission of sound waves in the range of 0.25–5 MHz, and it had broad frequency-matching characteristics. It was tested as a promising underwater packaging material. The experimental results showed that the encapsulated CMUT had good transmitting and receiving performance, and the center frequency did not shift. It also had a wide bandwidth, better directivity, higher receiving sensitivity and better receiving linearity. It was shown that sufficient signal amplitude and bandwidth could be obtained with the studied packing strategy.

A necessary step toward practical applications is to move from the laboratory electronics used in this work to the integrated electronics used in ultrasound system imaging. Preliminary tests of the prototype showed promising results as described in this paper. In addition, the matching layer had good stability in long-term testing, which is very important in practical application. The improvement and influence of different matching mechanisms on transducer frequency bandwidth, sensitivity and other acoustic properties have significant application values in ultrasonic medical imaging, underwater sensing and communication, industrial detection and other fields. Future work will include the development of a matching scheme with wide frequency/wide angle acoustic impedance matching performance, simplifying the process of fabricating a matching layer suitable for mass production and achieving cost savings.

## Figures and Tables

**Table 1:** Properties of PDMS and TiO_2_ particles.

Parameters	TiO_2_ (1)	PDMS (0)
$M^{\prime }$ (Pa)	3.69 × 10^11^	1.06 × 10^9^
$M^{\prime \prime }$ (Pa)	1.0 × 10^6^	4.63 × 10^6^
$G^{\prime }$ (Pa)	1.15 × 10^11^	1.0 × 10^6^
$G^{\prime \prime }$ (Pa)	1.0 × 10^6^	0.6 × 10^6^
ρ (kg m^−3^)	4260	1020
Radius R (nm)	<100	

**Table 2:** Structure parameters of CMUT.

Parameters	Values
Number of cells per device	900 (30 × 30)
Silicon membrane thickness/μm	2.83
Vacuum cavity diameter/μm	180
Vacuum cavity gap distance/μm	0.65
Oxide silicon insulating dielectric thickness/μm	0.15
Metal upper electrode diameter/μm	90
Metal upper and lower electrode thickness/μm	1
Silicon substrate thickness/μm	430

**Table 3:** Experiment results of unpackaged CMUT receiving sensitivity measurement.

Frequency/MHz	V_N_/mV	S_N_/(mV/MPa)	V_C_/mV	S_C_/(mV/MPa)	S_CdB_/dB
1	54.5	1206	483.7	10,702.86	−219.4
2	122.5	1126	1288.5	11,844.04	−218.5
3	245	1088	2153	9560.93	−220.4

**Table 4:** Experiment results of packaged CMUT receiving sensitivity measurement.

Frequency/MHz	V_N_/mV	S_N_/(mV/MPa)	V_C_/mV	S_C_/(mV/MPa)	S_CdB_/dB
1	221	1206	1596	8709.63	−221.2
2	264	1126	2188	9332.54	−220.6
3	445	1088	2580.7	6309.57	−224

## Data Availability

Not applicable.
